# Developmental Changes in Hypothalamic and Serum Oxytocin Levels in Prenatally Normally Nourished and Undernourished Rats

**DOI:** 10.3390/nu15122768

**Published:** 2023-06-16

**Authors:** Junki Imaizumi, Shuhei Kamada, Miyu Taniguchi, Tatsuro Sugimoto, Takaaki Maeda, Ryosuke Arakaki, Shota Yamamoto, Aya Shirakawa, Ayuka Mineda, Atsuko Yoshida, Takeshi Iwasa, Takashi Kaji

**Affiliations:** 1 Department of Obstetrics and Gynecology, Graduate School of Biomedical Sciences, Tokushima University, Tokushima 770-8503, Japan; t.m.j.imaizumi@gmail.com (J.I.); iwasa.takeshi@tokushima-u.ac.jp (T.I.); 2 Department of Renal and Genitourinary Surgery, Graduate School of Medicine, Hokkaido University, Sapporo 060-8648, Japan

**Keywords:** DOHaD, oxytocin, oxytocin receptor

## Abstract

Changes in the activities of some metabolic factors have been suggested to increase the risk of conditions associated with the Developmental Origins of Health and Disease (DOHaD). We examined changes in oxytocin (OT), a metabolic factor, and OT receptor (OTR) mRNA levels throughout the developmental period in rats of intrauterine undernutrition. Pregnant rats were divided into two groups: a maternal normal nutrition (mNN) and maternal undernutrition (mUN) group. Serum OT concentrations and hypothalamic mRNA levels of OT and OTR were measured in both offspring at various postnatal stages. Both offspring showed significant increases in serum OT concentrations during the neonatal period, significant reductions around the pubertal period, and significant increases in adulthood. Hypothalamic OT mRNA expression levels gradually increased from the neonatal to pubertal period and decreased in adulthood in both offspring. In the pre-weaning period, hypothalamic OT mRNA expression levels were significantly lower in the mUN offspring than in the mNN offspring. In the mUN offspring, hypothalamic OTR mRNA expression levels transiently increased during the neonatal period, decreased around the pubertal period, and increased again in adulthood, whereas transient changes were not detected in mNN offspring. These changes could affect nutritional and metabolic regulation systems in later life and play a role in the mechanisms underlying DOHaD.

## 1. Introduction

In recent years, the number of underweight young women has increased in Japan. Since the calorie intake of these women during pregnancy is not sufficient, and weight gain is low, nutritional deficiencies during pregnancy and their effects on the prenatal and neonatal development of their offspring are concerning. Body weight gain during pregnancy is lower in Japanese women than in women from other developed countries and results in full-term babies with lower average birth weight. Furthermore, undernutrition during pregnancy may lead to an increased risk of conditions that are associated with the Developmental Origins of Health and Disease (DOHaD) [1].

DOHaD is a pathophysiological hypothesis that suggests that fetal and/or neonatal nutritional disturbances, particularly prenatal undernutrition, increase the risk of chronic diseases in childhood and adulthood [2]. This hypothesis was originally based on the offspring of starved or undernourished pregnant women being at an increased risk of obesity and other metabolic diseases. Furthermore, babies affected by fetal growth restriction who experienced intrauterine undernutrition and grew rapidly in order to catch up to the normal growth process were subsequently shown to be at an increased risk of conditions associated with DOHaD.

Changes in the activities of some metabolic factors may increase the risk of conditions associated with DOHaD. Yura et al. reported transient increases in the level of leptin, an adipose-derived potent anorexigenic factor, during the neonatal to the prepubertal period (the leptin surge) in order to establish appropriate leptin activity and also showed that undernutrition in utero disrupted the leptin surge and consequently increased the risk of nutritional and metabolic diseases [3]. In addition to leptin, various factors, such as oxytocin (OT), serotonin, and ghrelin, have roles in nutrient metabolism [4,5]; however, it currently remains unclear whether these factors are involved in DOHaD. 

Among the hormones involved in nutrient metabolism, we focused on OT as the most important factor in relation to DOHaD. OT has been suggested to interact with leptin in order to appropriately regulate appetite and body weight because the administration of leptin has previously been shown to activate hypothalamic OT neurons in normal and diet-induced obesity rats [6,7]. OT is mainly produced in the hypothalamus: a brain region that plays an important role in the neuroendocrine regulation of energy metabolism [8]. OT, which is produced in magnocellular neurosecretory cells and in parvocellular neurosecretory cells, controls satiety, hunger, and the energy balance via the OT receptor (OTR) in both the paraventricular nuclei and supraoptic nuclei of the hypothalamus [9,10,11]. Previous studies have indicated the efficacy of OT in the treatment of obesity [12,13,14]. OTR-deficient mice exhibited late-onset obesity with increased amounts of visceral fat, and intraventricular injection of an OTR antagonist increased food intake by mice [15,16]. These findings suggest that endogenous OT plays a pivotal role in the prevention of obesity by regulating appetite. Exogenous OT has also been reported to reduce food intake by affecting the central appetite regulation system and promoting lipolysis in adipose tissue directly and indirectly through the OTR, resulting in a reduction in fat mass in experimental animals and humans [17]. In addition, OT exerts behavioral (e.g., confidence and cognition) and physical (e.g., muscle and bone formation) effects [18,19,20,21].

OT play a pivotal role in the regulation of nutrition and metabolism. If OT and OTR activities are induced by a transient increase in their levels during the early developmental period, as has been found for leptin, the disruption of this pattern by prenatal undernutrition may induce nutritional and/or metabolic disturbances and, thus, increase the risk of DOHaD. However, changes in OT or OTR levels in the early postnatal period and the effects of prenatal undernutrition have not yet been investigated. Therefore, we herein examined changes in OT and OTR levels throughout the developmental period and the effects of intrauterine undernutrition using experimental animals.

## 2. Materials and Methods

### 2.1. Animals

Pregnant Wistar rats were purchased (Charles River Japan, Tokyo, Japan) and kept individually at the University of Tokushima Animal Breeding House under controlled lighting (12 h light/dark cycle, lights on at 0800 and lights off at 2000) and temperature (24 °C) conditions. All animal experiments were performed in accordance with the ethical standards of the Animal Care and Use Committee of the University of Tokushima (T2021-25, date of approval: 1 June 2021). The animals were killed humanely at the end of the study. Pregnant rats were divided into two groups. In the maternal normal nutrition (mNN) group (*n* = 10), dams were allowed free access to water and standard chow (type MFG; Oriental Yeast Co., Ltd., Tokyo, Japan; 357 kcal/100 g, 13.6% of the calories provided were derived from fat, 25.7% from protein, and 60.7% from carbohydrates) during the gestation and lactation periods. In the maternal undernutrition (mUN) group (*n* = 10), dams were allowed approximately 50% of their daily feed intake, based on our previous findings [22], of the mNN group from day 14 of gestation until delivery and were allowed to feed ad libitum during lactation.

During pregnancy, maternal body weight changes were monitored in both the mNN and mUN groups and 24 h food intake was monitored in the mNN group. mNN and mUN offspring were weighed on the day of birth (defined as postnatal day (PND) 1). To control the litter size to 12–14 per dam, the pups were eliminated or moved to other dams and were fostered until weaning, depending on the total number of pups required for each experiment. In each experiment, each group of rats was used: 3–4 male rats and 3–5 female rats in each group on PND4-20, 10 male rats and 10 female rats in each group on PND28, 8 male rats and 7–8 female rats in each group on PND70. The pups were weighed at several time points after birth and were weaned on PND21. After weaning, the pups were kept three or four in a cage, and then one per cage after PND70. Male and female offspring were randomly selected from different litters for the experiments to avoid litter effects.

### 2.2. Experiment

At various postnatal stages, several pups from mNN and mUN offspring were randomly selected and killed by decapitation between 900 and 1100 h. Their trunk blood and whole brains were collected and used to measure serum OT concentrations, hypothalamic OT mRNA levels, and hypothalamic OTR mRNA levels. All surgical procedures were performed under sevoflurane-induced anesthesia.

### 2.3. Hormone Assay

Whole blood was centrifuged at 3000 rpm at 4 °C for 20 min, and serum samples were sent to a commercial laboratory (ASKA Pharmaceutical Medical Inc., Co., Ltd., Fujisawa City, Kanagawa, Japan), at which a chemiluminescent enzyme immunoassay was used to measure serum OT levels. The limit of detection for serum OT was 15 pg/mL, and the results obtained were not affected by vasopressin.

### 2.4. Quantitative Real-Time Polymerase Chain Reaction (PCR)

Whole hypothalamus explants were removed via an anterior coronal cut at the anterior margin of the optic chiasm, a posterior cut at the posterior margin of the mamillary body, a parasagittal cut along the hypothalamic fissure, and a dorsal cut 2.5 mm from the ventral surface. The total RNA was isolated from hypothalamic explants using a TRIzol^®^ reagent kit (Invitrogen Co., Carlsbad, CA, USA) and RNeasy^®^ mini kit (Qiagen GmbH, Hilden, Germany). cDNA was then synthesized with oligo (deoxythymidine) primers at 50 °C using the SuperScript III first-strand synthesis system for real-time PCR (Invitrogen Co.). PCR analysis was performed using the StepOnePlus™ real-time PCR system (PE Applied Biosystems, Foster City, CA, USA) and FAST SYBR^®^ green. The mRNA levels of OT and OTR were measured. The mRNA expression levels of each factor were normalized to the expression level of GAPDH. A dissociation curve analysis was also performed for each gene at the end of each PCR. Each amplicon generated a single peak. The relevant primer sequences, product sizes, and annealing temperatures are shown in Table 1. PCR conditions were as follows: initial denaturation and enzyme activation at 95 °C for 20 s, followed by 45 cycles of denaturation at 95 °C for 3 s, and annealing and extension for 30 s.

### 2.5. Statistical Analysis

All the results are presented as the mean ± standard error of the mean (SEM). In all statistical comparisons, *p* values < 0.05 were considered significant. Comparisons among mNN and mUN offspring were performed using Student’s *t*-test, the Mann–Whitney U test, or a one-way or two-way repeated measures analysis of variance (ANOVA) followed by the Tukey–Kramer test.

## 3. Results

### 3.1. Effects of Prenatal Undernutrition on Maternal and Offspring Body Weight

**Figure 1:**
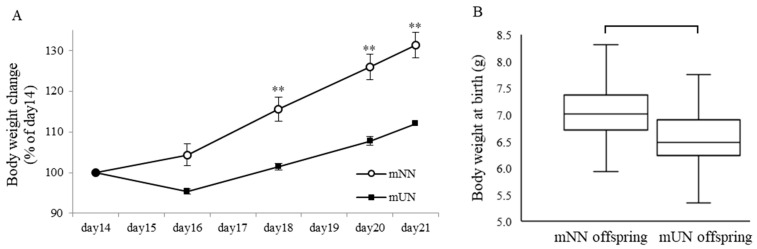

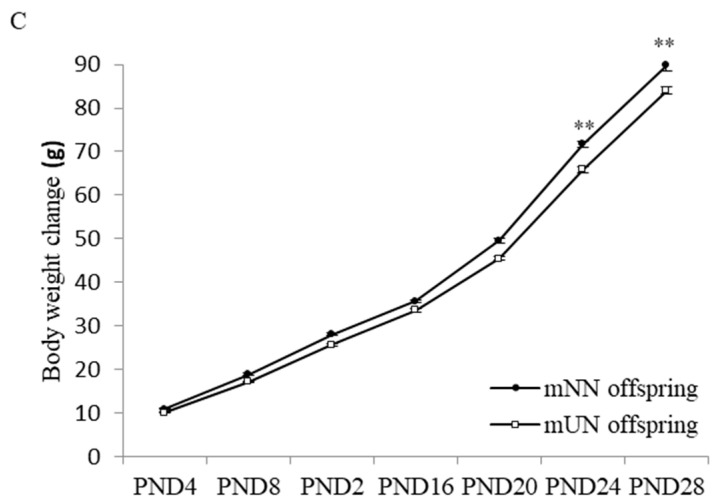
Maternal body weight change (**A**) during pregnancy in maternal normal nutrition (mNN) and maternal undernutrition (mUN) groups (*n* = 10 per group). In the mUN group, dams received 50% of the daily food intake of the mNN group from day 14. There were no significant differences in body weight between these two groups on day 14. Comparisons between the weights of the mNN and mUN offspring at birth (*n* = 103 per group) revealed that mUN offspring were significantly smaller than mNN offspring (**B**). Offspring’s body weight change. (**C**) in mNN offspring and mUN offspring (*n* = 53–61 per group). Body weight around PND 24 to 28 was significantly lower in the mUN offspring group than in the mNN offspring group. ** *p* < 0.01 vs. the mUN group at the same ages.

No significant differences were observed in the maternal body weight between the two maternal groups (the mNN and mUN groups) on day 14 (the initial day of feeding control). Body weight gain during pregnancy from gestation day 18 to 21 was significantly lower in the mUN group than in the mNN group (two-way ANOVA; *p* < 0.001). (Figure 1A) Body weight at birth was significantly higher for the mNN offspring than for the mUN offspring. (Figure 1B) No significant differences were observed in the offspring’s body weight between the two groups from PND4 to PND20. Body weight gain during PND 24 to 28 was significantly lower in the mUN offspring group than in the mNN offspring group (two-way ANOVA; *p* < 0.001). (Figure 1C) There were no significant differences during all the time points, but the weight of the mUN group was always lower than that of the mNN group. Although the graph is not shown, weight gain from PND 28 to 70 was always lower in the mUN offspring than in the NN offspring, and a difference in weight at day 70 for males was significantly lower in the mUN group than in the mNN group, but no significant difference in weight was observed for the females.

### 3.2. Changes in Serum OT Concentrations of Offspring

**Figure 2:**
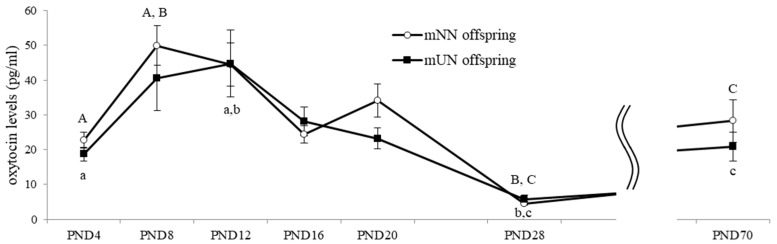
Serum oxytocin (OT) levels in maternal normal nutrition (mNN) offspring and maternal undernutrition (mUN) offspring (3–4 males and 3–5 females in each group on PND4-20, 10 males and 10 females in each group on PND28, and 8 males and 7–8 females in each group on PND70). A–C: data with same letters indicate significant differences (*p* < 0.05) within the mNN offspring. a–c: data with same letters indicate significant differences (*p* < 0.05) within the mUN offspring.

mNN and mUN offspring both showed significant increases in serum OT concentrations around PND8-12, more than doubling and a significant decrease at about one-tenth around PND28, followed by another significant increase around PND70. Although no significant differences were noted in prepubertal changes in the OT concentrations between mNN and mUN offspring, the timing of the early postnatal peak slightly differed between the mNN offspring (around PND8) and mUN offspring (around PND12) (Figure 2).

### 3.3. Changes in Hypothalamic OT and OTR mRNA Levels of Offspring

**Figure 3:**
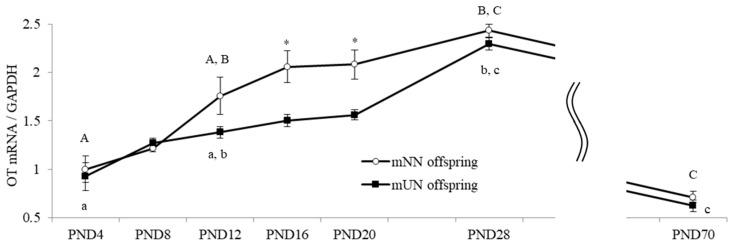
Hypothalamic oxytocin (OT) mRNA levels during neonatal development in maternal normal nutrition (mNN) offspring and maternal undernutrition (mUN) offspring (3–4 males and 3–5 females in each group on PND4-20, 10 males and 10 females in each group on PND28, and 8 males and 7–8 females in each group on PND70). Data on males and females were included. The relative expression of OT mRNA was assessed by dividing using the GAPDH expression, and the average mRNA expression level in mNN offspring on day 4 was expressed as 1.0. * *p* < 0.05 vs. the mUN group at the same ages. A–C: data with same letters indicate significant differences (*p* < 0.05) within the mNN offspring. a–c: data with same letters indicate significant differences (*p* < 0.05) within the mUN offspring.

Hypothalamic OT mRNA expression levels gradually increased from the neonatal to postnatal stages, i.e., from PND4 to PND28, and decreased on PND70 in the mNN and mUN offspring (Figure 3). Hypothalamic OT mRNA expression levels did not significantly differ between the mUN and mNN offspring from PND4-8, were slightly lower in the mUN offspring than in the mNN offspring from PND12-20, and were significantly lower by about 25% in the mUN offspring than in the mNN offspring on PND16-20 (*p* < 0.05). However, after PND28, similar hypothalamic OT mRNA expression levels were detected in both groups, with rapid reductions observed on PND70 (Figure 3).

**Figure 4:**
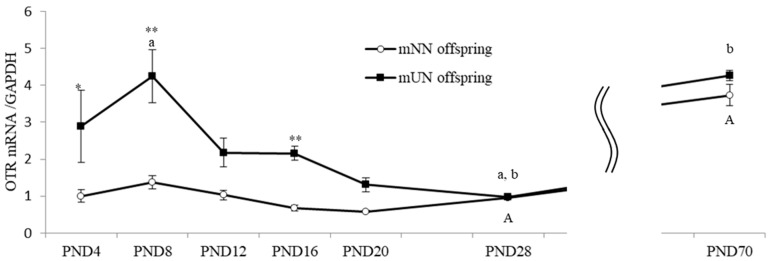
Hypothalamic oxytocin receptor (OTR) mRNA levels during neonatal development in maternal normal nutrition (mNN) offspring and maternal undernutrition (mUN) offspring (3–4 males and 3–5 females in each group on PND4-20, 10 males and 10 females in each group on PND28, and 8 males and 7–8 females in each group on PND70). Data on males and females were included. The relative expression of OTR mRNA was assessed by dividing using the GAPDH expression, and the average mRNA expression level in mNN offspring on day 4 was expressed as 1.0. ** *p* < 0.01, * *p* < 0.05 vs. mUN at the same ages. A: data with same letters indicate significant differences (*p* < 0.05) within the mNN offspring. a,b: data with same letters indicate significant differences (*p* < 0.05) within the mUN offspring.

Hypothalamic OTR mRNA expression levels in mUN offspring increased on PND8, decreased on PND28, and then increased again on PND70, whereas transient changes were not found in the mNN offspring (Figure 4). Hypothalamic OTR mRNA expression levels were significantly higher by about 2–3 fold in the mUN offspring than in the mNN offspring in the early postnatal period (PND4, 8, and 16; *p* < 0.05). However, hypothalamic OT mRNA expression levels in the mUN offspring gradually decreased and reached the same level as those in the mNN offspring on PND28 (Figure 4).

## 4. Discussion

In Japan, where a desire to be slim is prevalent among young women, there have been concerns that the conditions associated with DOHaD are becoming a serious issue for the future. Many studies that have focused on the hormonal and metabolic changes associated with DOHaD report the risks associated with undernutrition in offspring during pregnancy. Leptin, an adipocyte-derived appetite/metabolic regulator, is closely associated with DOHaD [23]. A previous study demonstrated transient increases in serum leptin levels from the neonatal to prepubertal period and also showed that maternal food restriction during pregnancy disturbed the leptin surge, resulting in leptin resistance and obesity in adulthood [3]. Namely, the maternal food restriction-induced premature onset of a transient leptin increase called a leptin surge can lead to pronounced body weight gain and increased fat mass in their offspring when fed with a high-fat diet [3]. Interestingly, these changes could be reproduced by exogenous leptin administration during the early neonatal period in offspring from the normally nourished dam. 

Leptin activates OT neurons in the hypothalamic PVN and POMC/CART neurons in the hypothalamic ARC via leptin receptors, whereas leptin inhibits AgRP/NPY neurons in the ARC. The activation of OT neurons activates POMC/CART neurons in the hypothalamic ARC nucleus, resulting in decreased food intake. Furthermore, the activation of oxytocin neurons is also associated with a negative feedback loop in the ARC nucleus in relation to AgRP/NPY. In addition to these pathways, oxytocin neurons control the posterior pituitary, whose activation leads to oxytocin secretion into the circulatory system: a pathway through which oxytocin reaches target tissues such as white adipose tissue. Thus, oxytocin and leptin secretion are closely related. During the neonatal period, undernourished rats experienced a decrease in their serum leptin levels. As a result of the reduced effect of leptin on the arcuate nucleus (ARC), the expression of NPY and POMC was altered. These changes in the body’s energy regulation system were referred to as leptin resistance and occurred early in development. This response contributed to DOHaD. In other words, if the variation in the oxytocin downstream of leptin is clarified, it could play a significant role in clarifying DOHaD [24,25]. 

OT and its receptor are also associated with a number of nutritional metabolism pathways and physiological processes, including parturition, lactation, cell proliferation, wound healing, and social behavior [26,27,28]. OT was recently shown to function as an appetite/metabolic regulatory factor, similar to leptin. The effects of OT on food intake, energy expenditure, and peripheral metabolism have been reported in many studies, and OT has attracted increasing attention in various therapeutic areas [29,30,31]. Since OT exerts strong anorexigenic effects, even on obese individuals, it has potential as an anti-obesity drug [13]. For example, the administration of OT or OT analogs reversed insulin resistance and glucose intolerance in high-fat-diet-induced obese mice. In addition, OT and OT analogs also improved insulin secretion and attenuated glucose intolerance in diabetic mice [13]. Similarly, we showed that the hypothalamic OT gene expression level and serum OT concentration were decreased in ovariectomized obese female rats and that body weight gain and food intake were attenuated by the administration of exogenous OT in these rats in a previous study [32]. Furthermore, we showed that the serum OT concentration decreased in androgen-induced obese PCOS model rats and that food intake was attenuated by the administration of exogenous OT [33].

We hypothesized that OT and OTR activities could initially be established by a transient increase in their levels during the early developmental period, similar to leptin and that this pattern could be disturbed by prenatal undernutrition. To the best of our knowledge, this is the first study to investigate the changes in OT and OTR levels throughout development and the effects of intrauterine undernutrition.

As we expected, the present results showed a transient increase in serum OT levels, namely, an OT surge, in the early postnatal period. The pattern of this OT surge in the present study was similar to that of the leptin surge. As noted above, the leptin surge plays an important role in establishing leptin activity and appropriately regulating appetite and metabolic functions. Similarly, previous studies have shown that neural projection pathways from specific hypothalamic areas, which regulate appetite and food intake can be permanently disrupted in leptin-deficient mice [34]. This study also suggested that leptin administration in adulthood could not reverse these alterations, whereas leptin treatment during the neonatal period restored neural development from this hypothalamic area. These data also support the hypothesis that the leptin surge plays a crucial role in the establishment of metabolic functions. In addition to leptin, gonadotropin levels transiently increased during the growth process, and these postnatal gonadotrophin surges could be associated with gonadal activation in the promotion of sexual maturation [35]. Hormone surges have been suggested to play pivotal roles in the growth process and establishment of normal physiological functions, and this may also be the case for the OT surge in the early postnatal period (PND4-8). However, further studies are needed to examine this hypothesis and elucidate the underlying mechanisms. 

In the present study, no significant differences were observed in serum OT levels between mNN and mUN offspring at any timepoint; however, the timing of the peak OT concentration was slightly later in mUN offspring than in mNN offspring. Since serum was collected every four days in the present study, the peak serum OT concentration may not have been accurately recorded. If samples had been collected every two days, clearer differences may have been detected between mNN and mUN offspring. A previous study reported that the peak of the leptin surge was also affected by prenatal undernutrition and that this change was associated with nutrient metabolism in later life in mice [3]. Therefore, if a shift in the timing of the OT surge occurred in mUN offspring, it may have resulted in nutrient and metabolism disturbances associated with DOHaD. Interestingly, previous studies have shown that the patterns of disturbance in the leptin surge are different between mice and rats. As noted above, leptin surges were advanced in mice derived from a food-restricted mother, while leptin surges were dramatically decreased in rats [36]. Again, OT surge was advanced, but not reduced, in rats derived from a food-restricted mother in the present study, suggesting that the effects of prenatal undernutrition on the change in neonatal leptin and OT may be different. One possibility is that leptin and OT, both of which are anorectic functions, might play a distinct function in the regulation of metabolic functions and also play different roles in the induction of DOHaD.

Regarding the present results on hypothalamic OT expression levels, the lack of a significant difference in OT expression levels between the mNN and mUN offspring from PND4-8 could have been due to the feeding volume in the early postnatal period being small and the production of anorexigenic factors, including OT, being suppressed to promote feeding. In contrast, the feeding volume gradually increased after PND12; therefore, OT mRNA levels may also have increased to regulate appetite. The induction of OT responses to the nutritional status was assumed to be normal in mNN offspring but could have been suppressed in mUN offspring. Consequently, undernutrition during the prenatal period caused a suppressed hypothalamic OT expression in the early postnatal period.

The present study revealed differences in the patterns of changes in serum OT concentrations and hypothalamic OT expression levels in mNN and mUN offspring. Therefore, OT can be released into the brain and peripheral circulation through separate and independent pathways, each with a different timescale [10]. It currently remains unclear whether peripheral OT levels in humans accurately reflect central OT activity [37]. Moreover, the present study found that changes in serum OT concentrations and hypothalamic OT expression levels from PND28 to PND70 were opposite to each other; i.e., serum OT levels increased, whereas hypothalamic OT levels decreased. Therefore, further studies are needed to clarify the underlying mechanisms and physiological implications of these discrepancies.

OTR activity has been shown to decrease with exposure to higher levels of OT [38]. The present results suggest that OT exposure in utero decreased hypothalamic OTR expression levels in the mNN group or that the reverse response occurred in the mUN group: namely, low OT exposure in utero increased these levels. This may be one of the compensatory responses used to overcome undernourished conditions during the prenatal period; however, its precise implications remain unclear. It may affect nutritional metabolism in later life.

The present study had a number of limitations. Although the quality of the immunoassay used to measure the serum OT levels was high, and we employed this method in our previous studies, the serum level of OT on PND 28 was markedly lower than the detection limit. Therefore, the reliability of the data obtained on PND 28 may be lower than that on other days. In addition, we were only able to collect 60–120 µL of blood per rat from PND4 rats by decapitation. Therefore, serum oxytocin concentrations in PND4 were measured by dilution, and the reliability of the data on PND4 may be lower than that on other days. Furthermore, since it was not possible to quantify the protein level of hypothalamic OT in this study due to technical limitations, it remains unclear whether the data obtained on hypothalamic gene expression levels reflect protein production. Moreover, whole hypothalamic blocks were used for the evaluations in the present study; therefore, subtle site-specific differences in OT and OTR mRNA levels may not have been detected. More precise experiments, e.g., evaluations using specific dissected nuclei, are needed to explain the present results. 

In summary, the present study has shown that serum OT levels transiently increase from the postnatal to the prepubertal period. In addition, prenatal undernutrition appeared to induce a temporary reduction in OT mRNA expression levels in the hypothalamus but a transient increase in OTR mRNA expression levels in the early postnatal period. These changes may affect nutritional and metabolic regulation systems in later life and be involved in the mechanisms underlying DOHaD. The present results provide insights into the developmental process for the endocrine mechanism of OT in the early postnatal period and the effects of fetal malnutrition and could contribute to the development of new drugs to treat oxytocin-related diseases. In addition, this research may alert Japanese youths of the dangers of a preference for thinness during pregnancy and the importance of nutritional recommendations.

It is important to note that species differences may exist in their reflection in humans. Therefore, further studies are needed to investigate whether similar mechanisms play a role in humans and to assess the validity of these findings.

## Figures and Tables

**Table 1:** Primer sequences, product sizes, and annealing temperatures.

Primer	Sequence	Annealing T (°C)
OT forward	GAACACAACGCCATGGCTGCCC	62
OT reverse	TCGGTGCGGCAGCCATCCGGGCTA	
OTR forward	CGATTGCTGGGCGGTCTT	67
OTR reverse	CCGCCGCTGCCGTCTTGA	
GAPDH forward	ATGGCACAGTCAAGGCTGAGA	70
GAPDH reverse	CGCTCGTGGAAGATGGTGAT	

## Data Availability

Not applicable.
